# Predictors of Limb Fat Gain in HIV Positive Patients Following a Change to Tenofovir-Emtricitabine or Abacavir-Lamivudine

**DOI:** 10.1371/journal.pone.0026885

**Published:** 2011-10-28

**Authors:** Allison Martin, Janaki Amin, Sean Emery, David Baker, Andrew Carr, David A. Cooper, Mark Bloch

**Affiliations:** 1 The Kirby Institute (formerly the National Centre in HIV Epidemiology and Clinical Research), University of New South Wales, Sydney, New South Wales, Australia; 2 East Sydney Doctors, Sydney, New South Wales, Australia; 3 St Vincent's Hospital and St Vincent's Centre for Applied Medical Research, Sydney, New South Wales, Australia; 4 Holdsworth House Medical Practice, Sydney, New South Wales, Australia; University of Cape Town, South Africa

## Abstract

**Background:**

Antiretroviral treatment (cART) in HIV causes lipoatrophy. We examined predictors of anthropometric outcomes over 96 weeks in HIV-infected, lipoatrophic adults receiving stable cART randomised to tenofovir-emtricitabine (TDF-FTC) or abacavir-lamivudine (ABC-3TC) fixed dose combinations.

**Methodology/Principal Findings:**

The STEAL study was a prospective trial of virologically suppressed participants randomised to either TDF-FTC (n = 178) or ABC-3TC (n = 179). Anthropometric assessment was conducted at baseline, weeks 48 and 96. The analysis population included those with baseline and week 96 data remaining on randomised therapy. Distribution of limb fat change was divided into four categories (≤0%, >0–10%, >10–20%, >20%). Baseline characteristics [demographics, medical history, metabolic and cardiovascular biomarkers] were assessed as potential predictors of change in percent subcutaneous limb fat using linear regression. 303 participants (85% of STEAL population) were included. Baseline characteristics were: mean (±SD) age 45 (±8) years; thymidine analogue nucleoside reverse transcriptase inhibitor (tNRTI) duration 4 (±3) years; limb fat 5.4 (±3.0)kg; body mass index 24.7 (±3.5) kg/m^2^. Mean (SD) limb fat gain to week 48 and 96 was 7.6% (±22.4) and 13.2% (±27.3), respectively, with no significant difference between groups. 51.5% of all participants had >10% gain in limb fat. Predictors of greater limb fat gain at week 96 were baseline tNRTI (10.3, p = 0.001), glucose >6 mmol/L (16.1, p = 0.04), higher interleukin 6 (IL-6) (2.8, p = 0.004) and lower baseline limb fat (3.8–6.4 kg – 11.2; >6.4 kg – 15.7, p trend<0.001).

**Conclusions/Significance:**

Modest peripheral fat gain occurred with both TDF-FTC and ABC-3TC. Baseline factors associated with more severe lipodystrophy (lipoatrophy, baseline tNRTI, raised IL6, and glucose) predicted greater limb fat recovery at 96 weeks.

## Introduction

Fat accumulation and depletion (lipoatrophy) are recognised as potential complications of antiretroviral therapy in HIV-infected patients [1], [2]. In particular, thymidine analogue nucleoside reverse transcriptase inhibitors (tNRTIs) and protease inhibitors are associated with potentially treatment limiting body fat redistribution manifestations [1]–[4]. These changes in association with adverse metabolic sequelae and adipose-related inflammation (known as lipodystrophy), may also lead to a higher risk of myocardial infarction [5]–[7].

In lipodystrophy there is adipocyte damage with an increase in tissue macrophages and production of inflammatory cytokines [8]. The inflammatory changes are also driven by alterations in adipocyte secreting hormones, particularly decreased adiponectin and leptin [9]. The reduced hormonal control results in an up-regulation of cytokines such as tumour necrosis factor (TNFα) and interleukin 6 (IL-6). With tNRTI-associated lipoatrophy there is depletion of adipocytes, causing reduction of adenosine 5′-triphosphate (ATP) production which affects lipid and glucose metabolism, and eventually leads to apoptosis and reduced fat cell mass [10].

Switching therapy from tNRTIs to non-thymidine based NRTIs such as abacavir (ABC) or tenofovir (TDF) has been shown to gradually reverse lipoatrophy, particularly in the more severe cases [11]–[16]. Recent studies have examined switching treatment to a fixed dose combination of Kivexa® (ABC/lamivudine (3TC)) or Truvada® (TDF/emtricitabine (FTC)), and reported these combinations to have similar efficacy [17], [18]. The STEAL study demonstrated that changing treatment to either ABC-3TC or TDF-FTC caused a gain in peripheral fat in both treatment groups [18].

The aim of this study was to examine baseline predictors of limb fat gain in the STEAL study population.

## Methods

### Objectives

To examine the predictors of anthropometric outcomes, assessed objectively via dual energy x-ray absorptiometry (DXA), in the STEAL study.

### Participants

Participants in the STEAL body composition sub-study were enrolled from the STEAL study clinical trial. The STEAL study was a 96-week, prospective, controlled trial of participants randomised to simplify existing NRTI drugs to either: tenofovir 300 mg-emtricitabine 200 mg (TDF-FTC) n = 178; or abacavir 600 mg-lamivudine 300 mg (ABC-3TC) n = 179. The primary STEAL study cohort and outcomes have been described [18]. Participants were recruited from 30 clinical sites around Australia.

### Description of Procedures or Investigations undertaken

DXA scans were performed at baseline, week 48 and week 96. Peripheral limb fat was described as absolute mass (kg) and percentage change from baseline to week 48 and 96. The distribution of limb fat mass changes at 96 were categorised as: ≤0%, >0–10%, >10–20% and >20%.

The baseline covariates that were analysed were: **Demographics** – age, gender, ethnicity, body mass index (BMI), smoking, blood pressure, concomitant medication; **HIV and antiretroviral therapy markers** – HIV duration, CDC category, CD4+ and CD8+ lymphocyte counts, duration of antiretroviral therapy (cART), use of tNRTI vs non-tNRTI, non-nucleoside reverse transcriptase inhibitors (NNRTI) vs protease inhibitors (PI), ABC vs TDF, continue on ABC or TDF vs switch from NRTI; **Body composition** – peripheral and trunk fat; **Metabolic markers** – total cholesterol, HDL cholesterol, LDL cholesterol, triglycerides; **Glycaemic markers** – glucose, HOMA (calculated assessment of insulin sensitivity), insulin; **Cardiovascular Biomarkers** – amyloid P, amyloid A, c-reactive protein (hsCRP), d-dimer, fibrinogen, soluble P selectin, vascular cell adhesion protein 1 (VCAM), intercelluIar adhesion protein 1 (ICAM), cystatin C, interleukin 6 (IL-6) and macrophage migration inhibitory factor (MIF-1). Information on methodology and coefficient of variation details for the assays used has been previously described [19].

### Ethics

The study was approved by each site's Research Ethics Committee (30 sites) and was registered at Clinicaltrials.gov (NCT00192634). Written informed consent was obtained from all participants in the sub-study.

### Statistical methods

The analysis was conducted on a per-protocol population defined by participants that had data from baseline and week 96 DXA scans and who remained on their randomised allocated treatment.

The percent change in limb fat mass was the primary endpoint and was compared by treatment arm (ABC-FTC vs TDF-3TC) using the T-test. Percent change in limb fat mass was also categorised into four groups: limb fat gain of ≤0%; 0.1–10%; 10.1–20%; and >20%. The distribution of participants in these categories according to treatment arm was compared using the Chi squared test.

The association between baseline covariates and percent change in subcutaneous limb fat from baseline to week 96 were analysed using linear regression. Multivariate models were built using forward stepwise methods. Predictors which achieved a p value <0.1 in univariate analysis were assessed for inclusion in the multivariate model. The final model was checked using backward elimination (data not shown). Baseline predictors were categorised (except for DXA) as previously described in the primary STEAL analysis and cardiovascular biomarker papers [18], [19].

Logistic regression was used to examined the relationship between the baseline characteristics and the greatest fat gain (>20% category) in the cohort.

## Results

The two randomised arms were well matched (Table 1) in regards to baseline characteristics, except the ABC-3TC treatment arm had a higher proportion of smokers then TDF-3TC (40 vs 29%). The mean baseline percent limb fat mass was 17.2 and 17.3%, ABC-3TC and TDF-FTC, respectively. This equates to an average baseline limb fat mass (SD) across the cohort of 5.4 (3.0) kg. Baseline characteristics for the per protocol population did not differ from those of the intention to treat population (data not shown).

**Table 1:** Baseline characteristics for HIV participants randomised to ABC-3TC or TDF-FTC (n = 357).

Baseline Characteristic	ABC-3TC	TDF-FTC
Age (years)	46±9	44±8
Male (%)	98	97
Ethnicity - white (%)	86	86
Body Mass Index (kg/m^2^)	24.7±3.5	24.8±3.6
HIV duration (years)	10±6	10±6
CD4+ count (cells/mm^3^)	627±306	599±257
Peripheral fat (%)	17.2	17.4
Trunk fat (%)	25.7	25.6
Current smoker (%)	40	29
Prior Abacavir (%)	20	21
Prior Tenofovir (%)	30	30
Prior Protease Inhibitor (%)	24	23
hs C-Reactive Protein (mg/L)	5.8±21.3	3.8±7.4
Interleukin-6 (pg/mL)	2.2±2.0	1.9±1.4
Amyloid P (ng/mL)	200±144	208±152
Amyloid A (ng/mL)	70±109	67±106
MIF-1 (pg/mL)**	2901±3109	2801±2979
D-Dimer (ngFEU/mL)	259±345	217±205
Fibrinogen (g/L)	2.8±0.8	2.7±0.7
P-selectin (ng/mL)	118±74	119±79
VCAM (ng/mL)***	392±201	424±223
ICAM (ng/mL)***	149±83	166±98
Cystatin C (mg/L)	0.8±0.1	0.8±0.1

*Results are expressed as mean ± SD or %.

**MIF-1 = macrophage migration inhibitory factor 1.

***VCAM = vascular cell adhesion molecule; ICAM = intercellular adhesion molecule.

The mean change in peripheral fat over 96 weeks was similar between treatment arms (p = 0.775). There was a 14.1% (5.9 kg) and 12.3% (6.0 kg) gain in peripheral fat for the ABC-3TC and TDF-FTC groups, respectively. The participants in both the ABC-3TC and TDF-FTC arms significantly increased limb fat mass over 96 weeks (p<0.001). The observed peripheral fat gain was clinically moderate with patients remaining technically “lipoatrophic”. Mean (SD) percent gain in limb fat mass for the entire cohort from baseline to week 48 and 96 was 7.4% (22.4) and 13.2% (27.3), respectively (see Figure S1). This is equivalent to an average peripheral fat gain of 224 g at week 48 and 487 g at week 96.

There was no significant difference (p = 0.493) between the treatment arms when comparing the proportion of percent fat change assessed using the four categories (Table 2). Within the entire cohort 34% of participants had no gain in peripheral limb fat. Fifty-one percent of participants had >10% gain in limb fat mass; ABC-3TC (52%) and TDF-FTC (51%), and 35% had >20% gain; ABC-3TC (37%) and TDF-FTC (33%).

**Table 2:** Percent change in peripheral limb fat mass from baseline to week 96 expressed as quartile categories, based on randomised arm (n = 303).

Randomised Arm	≤0%	>0–10%	>10–20%	>20%
TDF-FTC (n)*	55	21	28	52
ABC-3TC (n)*	49	22	21	55
Total n (%)	104 (34)	43 (14)	49 (16)	107 (35)

*TDF-FTC = tenofovir - emtricitabine; ABC-3TC + abacavir – lamivudine.

*p value comparing arms over 96 weeks p = 0.493.

Covariates associated with the percent increase in peripheral fat mass over 96 weeks on univariable analysis are summarised in Table 3. The multivariate linear regression analysis demonstrated that the baseline covariates that were significantly and positively associated with greater limb fat gain were baseline thymidine nucleoside therapy (coefficient 10.3, p = 0.001), fasting glucose >6 mmol/L (coefficient 16.1, p = 0.04), higher IL-6 (coefficient 2.8, p = 0.004) and lower baseline peripheral limb fat (3.8–6.4 kg - coefficient 11.2; >6.4 kg - coefficient 15.7, p trend<0.001). Baseline use of tenofovir or abacavir did not predict fat gain. The adjusted R squared for this model was 0.15.

**Table 3:** Baseline covariates assessed in the multivariate model of percent change in peripheral limb fat mass at 96 weeks for HIV participants randomised to ABC-3TC or TDF-FTC (n = 303).

Baseline Variable	Categories	Univariate Analysis				Multivariate Analysis			
		Coefficient	Lower 95%CI	Upper 95%CI	P	Coefficient	Lower 95%CI	Upper 95%CI	P
On tNRTI*		12.4	6.3	18.5	<0.0001	10.3	4.4	16.2	0.001
tNRTI duration*		0.1	0.04	0.2	0.003	−0.03	−0.1	0.1	0.451
Stratification	Abacavir (reference group)				0.016				0.071
	Tenofovir	4.3	−4.6	13.2		7.9	−0.7	16.5	
	Other	11.0	3.0	19.1					
Interleukin-6		3.3	1.2	5.3	0.002	2.8	0.9	4.8	0.004
Total Cholesterol (mmol/L)	≤5.5 (reference group)				0.039				0.074
	>5.5	−6.7	−13.1	0.4		−5.5	−11.6	0.5	
Impaired fasting glucose/diabetes (mmol/L)	≤6 (reference group)				0.067				0.040
	>6	15.4	−1.1	31.8		16.1	0.7	31.5	
Peripheral Fat (kg)	<3.8 (reference group)				<0.001				<0.001
	3.8 to 6.4	−10.9	−18.2	−3.7		−11.2	−18.3	−4.1	
	>6.4	−17.5	−24.8	−10.2		−15.7	−22.8	−8.6	
Trunk fat (kg)	<7.99 (reference group)				0.010				0.869
	7.99 to 11.51	−5.2	−12.6	2.3		−0.3	−7.8	7.2	
	>11.51	−9.9	−17.4	−2.4		−0.8	−9.8	8.3	

*tNRTI = thymidine nucleoside reverse transcriptase inhibitors.

The multivariate logistic analysis of the baseline predictors of peripheral fat gain in the participants (35% of cohort) that experienced >20% gain were prior tNRTI use (OR 2.2, p = 0.001) and lower peripheral fat (OR 0.42, p = 0.06).

## Discussion

In previous studies various markers have been investigated to predict the development of lipoatrophy [8]–[10], however very few covariates have been tested as predictors of fat gain associated with reversal of lipoatrophy [11]. This study demonstrated that similar fat mass gains were evident with both TDF-3TC and ABC-FTC in virologically stable, NRTI pre-treated HIV positive patients. At baseline the average limb fat mass across the cohort was 5.4 kg, which corresponds to a mildly lipodystrophic population. More than 50% of participants gained at least 10% peripheral body fat during the 96 week follow-up. In this analysis we identified four covariates that were independent predictors of greater limb fat gain following the switch to either treatment arm: baseline regimen containing a tNRTI, lower peripheral fat, impaired fasting glucose (>6 mmol/L), and higher IL-6. The composite model including these variables explained 15% of the observed changes in body fat over 96 weeks.

Antiretroviral switch studies have previously demonstrated a gain in limb fat after switching from tNRTIs to ABC [11]–[13]. Martin et al. demonstrated a 2.5 times greater increase in limb fat mass when patients on an tNRTI were switched to ABC (1.26 kg) treatment compared to zidovudine or stavudine (0.49 kg) over 104 weeks [11]. McComsey et al. reported a 22% and 18% increase in arm and leg fat respectively, after switching from stavudine to ABC [12]. Similarly, studies examining switching therapy from tNRTI to TDF have demonstrated increases in total fat mass by 21% [14] and limb fat mass of 0.38 kg [15]. One other study compared switching to either TDF or ABC and reported similar limb fat gain in both treatment arms over 48 weeks, increasing by 0.33 kg (TDF) and 0.48 kg (ABC) [16]. Therefore, the limb fat increase reported in the STEAL study is comparable to previous studies.

Reported predictors for the development of lipoatrophy include increased age, being white race, increased duration of HIV infection, pre-treatment low nadir CD4+ T lymphocyte count and high plasma HIV RNA load, previous AIDS-defining illness, hepatitis C co-infection, use of a tNRTI (particularly stavudine), increased duration of NRTI therapy, adherence to cART, and TNFα polymorphism 238 G/A [3], [20]–[23]. Only one previous study has assessed predictors of the reversal of lipoatrophy and found baseline BMI to be associated with greater fat gain after switching from tNRTIs to ABC [11].

We have found that baseline tNRTI usage and low peripheral limb fat were strongly correlated with greater limb fat gain over 96 weeks. Therefore, those with more severe lipoatrophy, probably caused by tNRTI, were more likely to experience a greater gain in limb fat after switching to ABC or TDF. This could be due to a causal link or could simply be a regression to the mean effect: for example those with less fat at baseline are more likely to gain fat throughout the study; and vice versa those with higher baseline limb fat did not have lipoatrophy and therefore experienced no limb fat gain.

We also found that impaired glucose metabolism (impaired fasting glucose) at baseline predicted greater fat gain. These results suggest there may be an indirect effect of NRTIs on glucose metabolism via the adipose tissue changes caused by NRTIs [24]. However, insulin resistance, as measured indirectly by HOMA or insulin levels did not. Therefore, these results may also be a result of a type-2 error.

Adipose tissue has been shown to produce inflammatory cytokines, such as C-reactive protein and IL-6 [25]. Some studies demonstrated high IL-6 to be correlated with limb fat in HIV patients with lipodystrophy [26], [27], whereas others have only demonstrated the same in HIV positive patients compared with controls, not those with lipodystrophy [28]. IL-6 mRNA expression has also been shown to increase in peripheral adipocytes in HIV treated participants with lipodystrophy [8]. The finding in this study that higher IL-6 levels at baseline was associated with greater fat gain over 96 weeks, is consistent with results from these previous studies suggesting that high IL-6 may represent those participants with lipodystrophy at baseline. This finding also reinforces the observation that there are immunological abnormalities that associate with lipodystrophy.

### Limitations

Limitations of this study relate to the inclusion of a HIV population that is not representative of the wider HIV international community i.e. participants were predominantly Caucasian men recruited within Australia, with well controlled viraemia. In addition, 20% and 30% of our participants had previously been exposed to abacavir and tenofovir, respectively. Therefore these data cannot be generalised beyond similar populations. Stratification was conducted on previous NRTIs to control for this confounder. There was a low predictive value (15%) of the regression equation suggesting that factors other than those measured may play an important role in changes in peripheral fat. The characteristic with the strongest association with change in peripheral fat was baseline limb fat which may suggest a regression to the mean affect.

This study shows that changing to either ABC or TDF is associated with partial, but probably limited, reversal of lipoatrophy over 96 weeks. The fat gain may be predicted by baseline tNRTI, low peripheral fat, impaired glucose metabolism and high IL-6. These factors associated with predicting limb fat recovery could represent the patients with severe lipodystrophy and the associated inflammatory response. This may also infer a simple regression to the mean effect for patients with more severe lipodystrophy. Further studies into biomarkers should be conducted to assess prediction models of fat gain, most importantly those that are associated with bone and body composition.

## Supporting Information

Figure S1 **Absolute and percentage change from baseline in peripheral limb fat mass to week 48 and 96 for the entire cohort of STEAL participants (ABC-3TC and TDF-FTC) n = 303.** *p<0.001 of change from baseline to week 96.
(TIF)
