# Supplementary figures and images for: Predictors of Limb Fat Gain in HIV Positive Patients Following a Change to Tenofovir-Emtricitabine or Abacavir-Lamivudine
Source: PLoS One. 2011 Oct 28;6(10):e26885. doi: 10.1371/journal.pone.0026885 (PMC3203920; doi:10.1371/journal.pone.0026885)

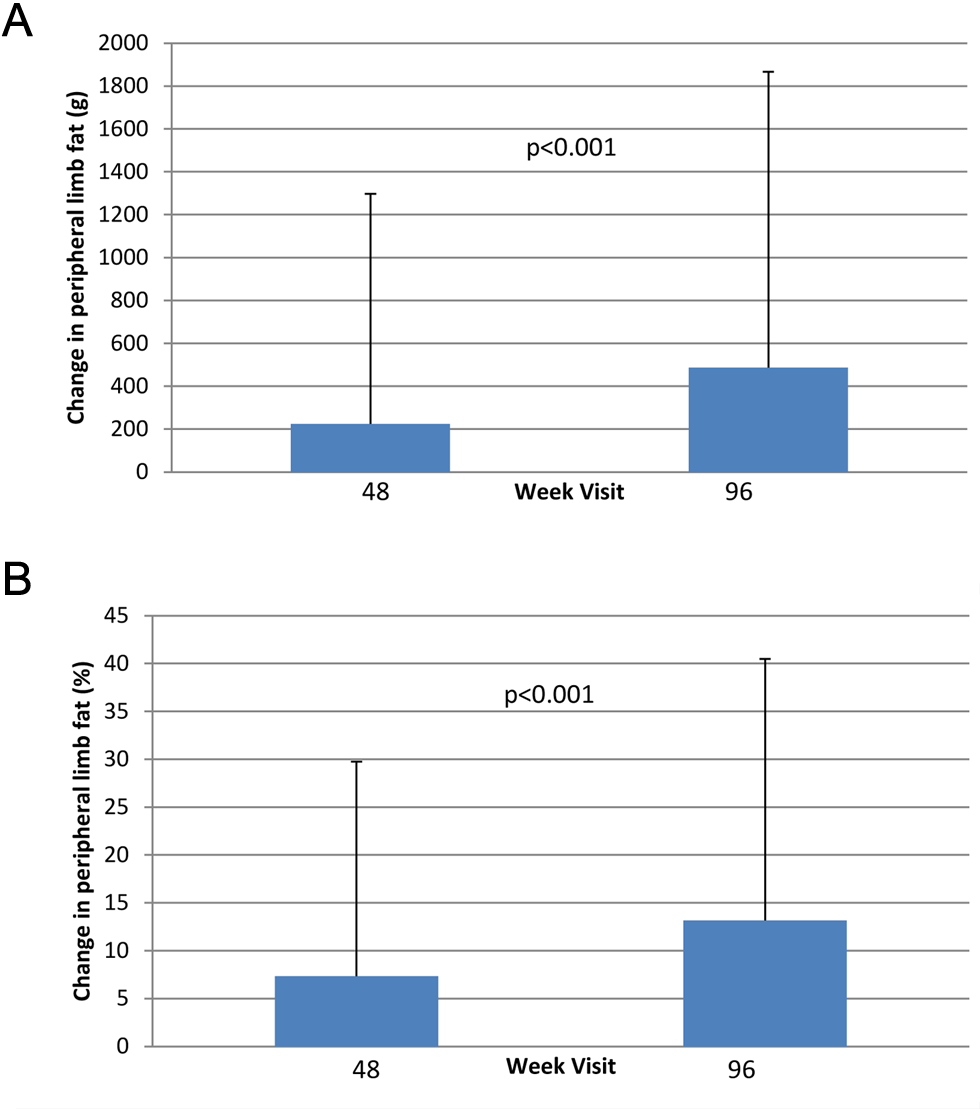

Supplement: Figure S1 — Absolute and percentage change from baseline in peripheral limb fat mass to week 48 and 96 for the entire cohort of STEAL participants (ABC-3TC and TDF-FTC) n = 303. *p<0.001 of change from baseline to week 96. (TIF) [file pone.0026885.s001.tif]
